# Complete genome sequence of *Novosphingobium* sp. strain BL-52-GroH shows unusual dual chromosome architecture

**DOI:** 10.1128/mra.01304-24

**Published:** 2025-04-28

**Authors:** Jonathan Seid, Paul D. Boudreau

**Affiliations:** 1 Department of BioMolecular Sciences, School of Pharmacy, University of Mississippi 551786 , University, Mississippi, USA; University of Strathclyde, Glasgow, United Kingdom

**Keywords:** *Novosphingobium*, sphingolipids, dual chromosomes

## Abstract

*Novosphingobium* sp. BL-52-GroH was isolated from soil at the University of Mississippi campus. This strain’s nanopore-sequenced genome showed two circular megabase-length chromosomes and two plasmids. Annotation showed housekeeping genes on both chromosomes, marking this *Novosphingobium* strain as another example in the genus with dual chromosome architecture.

## ANNOUNCEMENT

We are isolating novel strains from the sphingolipid-producing Sphingomonadales (1). Here, we report the genome of a *Novosphingobium* strain found with our PCR assay for the serine palmitoyltransferase gene (2). Our assembly resulted in four circular contigs, two 1.79 and 3.55 Mb chromosomes, and two 500 and 543 kb plasmids, an unusual architecture versus the single circular chromosome in most bacteria (3), but one observed before in this genus (4, 5).

BL-52-GroH was isolated from soil collected next to a trash can on the University of Mississippi Grove (34°21'54.5" N 89°31'58.0" W); 1 g of soil was vortexed with 5 mL of phosphate-buffered saline (6) for 15 min, and then, the unfiltered sample was diluted 100× and struck onto an agar plate of defined medium for siderophores (DMS)-pyruvic acid (7), treated with nystatin (Alfa Aesar). A colony restruck on a fresh DMS-citric acid plate produced pure colonies with a yellow-colored, circular, flat, and entire morphology. A pure colony was picked into 5 mL of LB Lennox broth (Fisher Bioreagents) and incubated on a shaker at moderate speed for 48 h at room temperature in air (ca. 23°C, 1 atm). We vortexed 1:1 turbid culture to sterile 50% glycerol to prepare a −70°C stored stock.

Genomic DNA was isolated from cultures revived from stock: For 16S sequencing (Genewiz’s 16S rRNA Service; New Jersey, USA), we used the E.Z.N.A. Bacterial DNA kit with the optional bead-beating (Omega Bio-Tek) and separately for genome sequencing using approximately 10 µL of pelleted cells with the NucleoBond High Molecular Weight (HMW) DNA Enzymatic Lysis protocol (Macherey-Nagel) with 10 µL of lysozyme (Omega Bio-Tek). HMW DNA was quantified by fluorescence on a Qubit, then concentrated with SeraMag beads, before vendor (Plasmidsaurus; Kentucky, USA) nanopore sequencing. The vendor used V14 library prep chemistry for R10.4.1 cells on a PromethION with Dorado basecalling on super-accuracy mode to produce 162,406 raw .fastq genomic reads, with an N50 of 9,215 bp.

In Geneious (version 2023.0.4), the raw 16S forward/reverse Sanger reads were trimmed (error probability limit of 0.005) and pairwise aligned with each other. Then, in Geneious, a Megablast search of the consensus found a 99.5% pairwise hit to *Novosphingobium* sp. NJ-NJ2-1019 (MK863544) in the nucleotide collection (nr/nt) (8, 9). Raw genomic reads were processed with FiltLong (version 0.2.1) (10), removing reads below 500 bp then to keep the top 98% of reads by quality score, and separately with cutoffs at 1,000 bp and the top 90%. The 500 bp/98% file was assembled using Flye (version 2.9-b1778) with the nanopore high-quality setting (11), producing an assembly of four circular contigs (x127 mean coverage). Polishing the assembly with the 1,000 bp/90% file by Racon (version 1.5.0) (12) and then Medaka (version 1.7.2) (13) produced the final 6,384,257 bp genome with 65.9% GC content. The NCBI Prokaryotic Annotation Pipeline annotation (version 6.8, by GenBank) (14) showed the chromosomes shared housekeeping genes (e.g., rRNA genes). OrthoANI Tool (OAT, version 0.93.1) comparison to all *Novosphingobium* genomes on GenBank with complete or chromosome level assemblies suggested that BL-52-GroH is a novel strain (Table 1) (15).

**TABLE 1:** The OrthoANI tool (OAT) comparisons against BL-52-GroH

GenBank reference	OAT %Identity vs. BL-52-GroH	Scientific name	Assembly
GCA_000876675.2	82.38%	*Novosphingobium* sp. P6W	ASM87667v2
GCA_001742225.1	81.19%	*Novosphingobium resinovorum*	ASM174222v1
GCA_021227995.1	81.14%	*Novosphingobium kaempferiae*	ASM2122799v1
GCA_030369695.1	80.80%	*Novosphingobium resinovorum*	ASM3036969v1
GCA_017309955.1	79.28%	*Novosphingobium* sp. KA1	ASM1730995v1
GCA_009707465.1	78.28%	*Novosphingobium* sp. Gsoil 351	ASM970746v1
GCA_035658495.1	77.91%	*Novosphingobium* sp. RL4	ASM3565849v1
GCA_044029535.1	77.49%	*Novosphingobium* sp. BL-8H	UM_Novm8H_1.0
GCA_044029435.1	77.30%	*Novosphingobium* sp. BL-8A	UM_Novo8A_1.0
GCA_000253255.1	76.96%	*Novosphingobium* sp. PP1Y	ASM25325v1
GCA_000767465.1	76.89%	*Novosphingobium* *pentaromativorans* US6-1	ASM76746v1
GCA_025340265.1	76.75%	*Novosphingobium* sp. 9	ASM2534026v1
GCA_018417475.1	76.14%	*Novosphingobium* *decolorationis*	ASM1841747v1
GCA_005145025.1	74.19%	*Novosphingobium* sp. EMRT-2	ASM514502v1
GCA_037076535.1	73.98%	*Novosphingobium olei*	ASM3707653v1
GCA_000013325.1	73.91%	*Novosphingobium* *aromaticivorans* DSM 12444	ASM1332v1
GCA_003454795.1	73.66%	*Novosphingobium* sp. THN1	ASM345479v1
GCA_007954425.1	73.64%	*Novosphingobium ginsenosidimutans*	ASM795442v1
GCA_015169775.1	73.31%	*Novosphingobium* sp. ES2-1	ASM1516977v1
GCA_036784765.1	73.09%	*Novosphingobium* sp. ZN18A2	ASM3678476v1
GCA_034424435.1	73.06%	*Novosphingobium capsulatum*	ASM3442443v1
GCA_028607105.1	72.13%	*Novosphingobium humi*	ASM2860710v1
GCA_030388345.1	71.99%	*Novosphingobium humi*	ASM3038834v1
GCA_028736195.1	71.91%	*Novosphingobium* sp. KACC 22771	ASM2873619v1

## Data Availability

The final annotated *Novosphingobium* sp. BL-52-GroH genome, and Sequence Read Archive of the raw vendor reads, are available on GenBank under the BioProject archive as PRJNA1164209, with the assembly accession number ASM4481308v1, and of the read archive SRR31290250. The preliminary 16S sequence has also been uploaded to GenBank under the accession number PV107391.
